# Treatment of Allergic Rhinitis With Desloratadine: Results of a Multinational Observational Study in the Middle East Gulf Region

**DOI:** 10.1097/WOX.0b013e31822a6e9a

**Published:** 2011-08-15

**Authors:** Tamer MH Adham

**Affiliations:** 1 Faculty of Medicine, Ain Shams University, Egypt, Al Noor Hospital, Abu Dhabi, United Arab Emirates

**Keywords:** allergic rhinitis, desloratadine, Middle East, nasal congestion, peak nasal inspiratory flow

## Abstract

**Background:**

Allergic rhinitis (AR) affects up to 36% of the population in the Middle East Gulf States. The second-generation nonsedating antihistamine desloratadine has demonstrated safety and efficacy in the treatment of AR; however, few studies have evaluated this agent in Arab and Asian populations in the Middle East.

**Methods:**

This open-label study enrolled subjects ≥ 12 years with moderate-to-severe AR; they received desloratadine 5 mg QD for 2 weeks. Endpoints included change in mean individual nasal and ocular symptom scores, total symptom score (TSS), and peak nasal inspiratory flow (PNIF) and percentage improvement in global response to therapy.

**Results:**

There were 602 subjects from 5 Middle East countries enrolled. After 2 weeks, desloratadine significantly (*P *< 0.0001) reduced mean scores for individual nasal and total ocular symptom scores and TSS. PNIF measures of nasal congestion were significantly (*P *< 0.0001) improved after treatment. Most subjects obtained complete (38.1%) or marked (47.2%) relief of AR symptoms. Treatment failure was reported in 2.2% of subjects. No adverse events were reported, and no subjects discontinued treatment.

**Conclusion:**

Most subjects reported significant symptom relief with desloratadine 5 mg/d for 2 weeks. Desloratadine is effective in the treatment of AR in Arab and Asian subjects in the Middle East Gulf region.

## 

Allergic rhinitis (AR), characterized by rhinorrhea; nasal itching; nasal congestion; sneezing; red, itching, watery eyes; and/or itching ears or palate, has become increasingly prevalent in the Middle East Gulf region; estimates indicate that up to 36% of the region's population may be affected [1-3]. Rapid development, the oil industry, and modernization have led to pollution and the introduction of non-native species of plants and grass [1,3-5] and amenities such as carpeting and air conditioning, contributing to an overall increased sensitivity to allergens [6,7].

Patient surveys reveal that the symptoms of AR, particularly nasal congestion, have a marked impact on quality of life,[8,9] emotional well-being,[9,10] work or school productivity,[11-13] and sleep [9,14]. In 2 large surveys of patients with AR, nasal congestion was the most bothersome symptom and was reported to affect sleep,[9] to impair work productivity, and to increase absenteeism [8,9]. In a third survey, adult participants who reported nasal congestion resulting from allergy were 1.8 times more likely to have moderate to severe sleep-disordered breathing than those without nasal congestion because of allergy [14]. Further, students in the United Kingdom, 15 to 17 years of age, with symptoms of AR were 40% more likely to drop a grade between their practice and final examinations than were students who were symptom-free [15].

The Allergic Rhinitis and its Impact on Asthma (ARIA) working group recommends second-generation nonsedating antihistamines as a first-line therapy for AR [16]. Desloratadine, a potent nonsedating second-generation antihistamine, is indicated for the treatment of AR in adults and adolescents ≥ 12 years irrespective of duration (intermittent/persistent) or seasonality (seasonal/perennial) [17]. Numerous clinical [18-25] and observational studies [26-37] have found that desloratadine treatment improves the classic symptoms of seasonal (SAR), perennial (PAR), intermittent (IAR), and persistent AR (PER), including nasal congestion. Desloratadine is well tolerated with a safety profile similar to placebo [18-20].

This observational study evaluated the efficacy of desloratadine 5 mg once daily in improving nasal and ocular symptom scores and nasal airflow as measured by peak nasal inspiratory flow (PNIF) in Arab and Asian subjects ≥ 12 years with SAR or PAR in a "real-world" clinical setting in 5 Middle East countries.

## Methods

This open-label, noninterventional, practice-based study was conducted at 47 centers in the Middle East countries of Bahrain, Kuwait, Oman, Qatar, and United Arab Emirates (UAE). The study was performed in accordance with the Declaration of Helsinki and the guidelines of Good Clinical Practice. Written informed consent was obtained from each subject.

Eligible subjects were 12 years or older and had a ≥ 2-year history of SAR or PAR, confirmed by standard skin prick test with the following allergens: *Dermatophagoides pteronyssinus, Dermatophagoides farinae*, cockroach, *Asperigillus*, yeast, weed mix, and cat and dog fur.

A baseline total symptom score (TSS, sum of nasal discharge/rhinorrhea, nasal congestion, sneezing, nasal itching, and ocular symptoms) of ≥ 8, a nasal congestion score of ≥ 2, an ocular symptom score of ≥ 2 on a 4-point scale (0 = none, 3 = severe), indicative of moderate-to-severe disease, were also required. TSS is widely used in clinical studies of allergic rhinitis.

Subjects were excluded from the study if they were pregnant or nursing; had another clinically significant disease, particularly nasal disorders or structural abnormalities that might interfere with nasal airflow; had current or a history of chronic sinusitis or purulent postnasal drip; or had sensitivity to the study drug or its excipients. Subjects with asthma requiring chronic use of inhaled or systemic steroids were also excluded.

Subjects received oral desloratadine 5 mg once daily for 14 days, in accordance with the approved product labeling and current medical practice. No concomitant medications for the treatment of AR were permitted. Investigators evaluated symptom severity at baseline (day 0 to the first day of dosing) and at the end of treatment. PNIF was measured 3 times before and after treatment with the best reading at each visit recorded using a portable, hand-held inspiratory flow meter (30-370 L/min; In-Check Nasal; Clement Clarke International, Essex, United Kingdom). At the end of treatment, physicians and subjects jointly rated the global response to therapy using a 5-point scale (0 = no relief, treatment failure; 1 = slight relief; 2 = moderate relief; 3 = marked relief; 4 = complete relief).

Study end points included subjective change from baseline in mean individual nasal symptom scores and mean ocular symptom score, objective change from baseline in the mean PNIF, and the physician/subject assessment of global response to therapy.

Data were analyzed on an intent-to-treat basis using the Wilcoxon matched-pairs signed rank test. Student *t *test and analysis of variance (ANOVA) were used for continuous variables; *χ*^2 ^tests were used for categorical variables. The significance level was set at ≥ 0.05.

## Results

**Figure 1:**
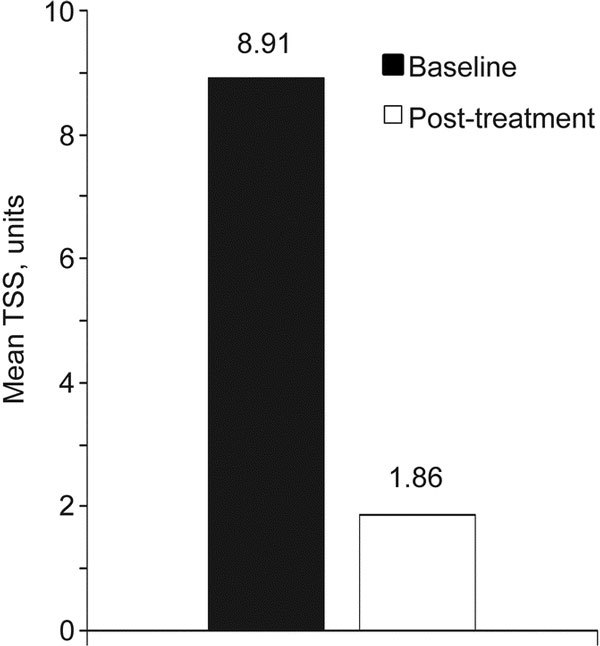
**Mean change from baseline to end of desloratadine treatment in TSS**. *P *< 0.0001 (ANOVA). TSS, total symptom score.

**Figure 2:**
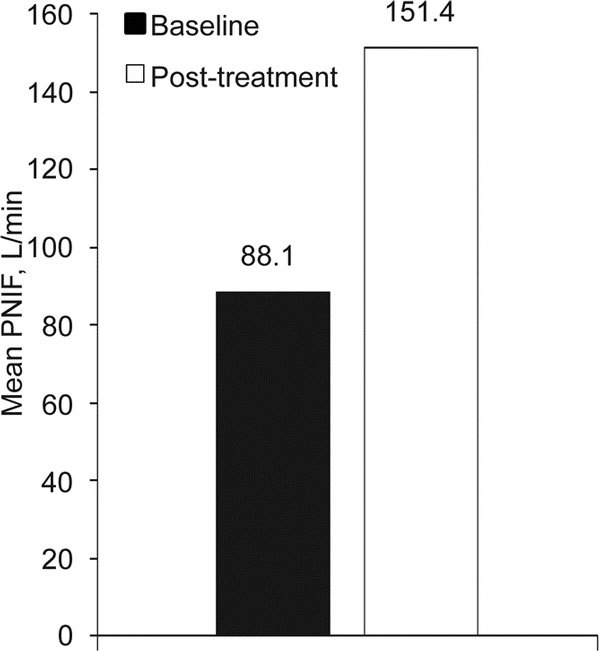
**Mean change from baseline to end of desloratadine treatment in PNIF (L/min)**. *P *< 0.0001 (ANOVA). PNIF, peak nasal inspiratory flow.

A total of 602 subjects were enrolled. Demographics and baseline clinical characteristics are shown in Table 1. Treatment with desloratadine significantly (*P *< 0.0001) reduced mean scores from baseline for individual nasal and ocular symptoms (Table 2). The mean TSS was significantly (*P *< 0.0001) reduced from baseline after 2 weeks of treatment (Figure 1). Nasal airflow measured by PNIF increased significantly (*P *< 0.0001) after desloratadine therapy, with a 72% mean improvement from baseline (Figure 2).

**Table 1:** Baseline Demographics and Clinical Characteristics

Characteristics	
Age, year (SD)	33.7 (12.1)
Sex, n (%)	
Male	364 (60.5)
Female	213 (35.4)
Unrecorded	25 (4.1)
Race, n (%)	
Arab	295 (49.0)
Asian	224 (37.2)
Other	19 (3.2)
Unrecorded	64 (10.6)
Diagnosis, n (%)	
PAR	217 (36.0)
SAR	363 (60.3)
Unrecorded	22 (3.7)
Mean clinical characteristics (SD)	
TSS	8.9 (2.9)
Nasal congestion	2.2 (0.75)
PNIF, L/min	88.1 (52.5)

PAR, perennial allergic rhinitis; PNIF, peak nasal inspiratory flow; SAR, seasonal allergic rhinitis; TSS, total symptom score.

**Table 2:** Mean Percentage Change Between Baseline and Posttreatment Individual Symptom Scores

Symptom	Mean Baseline Score (SD)	Mean Posttreatment Score (SD)	Mean Percentage Change From Baseline, %
Rhinorrhea	2.01 (0.76)	0.44 (0.56)*	78.1
Nasal congestion	2.16 (0.75)	0.62 (0.65)*	71.3
Sneezing	1.93 (0.79)	0.35 (0.55)*	81.9
Nasal pruritus	1.74 (0.89)	0.30 (0.53)*	82.7
Ocular symptoms	1.23 (0.96)	0.17 (0.43)*	86.2

*P < 0.0001 (ANOVA), baseline versus posttreatment score.

For 564 subjects for whom treatment response was recorded, symptom relief at the end of treatment was complete (38.1%), marked (47.2%), or moderate (12.6%) after joint assessment by physicians and subjects. Treatment failure occurred in 2.2% of subjects who reported slight (1.8%) or no (0.4%) relief. Global therapeutic response for 38 subjects (6.3%) was not recorded, and these subjects were considered treatment failures for the intent-to-treat analysis. Desloratadine treatment was well tolerated. No adverse events were reported during the study, and no subjects discontinued treatment.

## Discussion

After 14 days of treatment, desloratadine therapy provided significant improvement from baseline in total and individual symptom scores and in nasal airflow measured by PNIF for Arab and Asian subjects residing in 5 Middle East Gulf countries. Furthermore, global therapeutic response was reported as complete, marked, or moderate in 97.9% of subjects.

The improvement in subjective symptom scores and therapeutic response found in this open-label study supports results from earlier observational safety studies,[26-37] placebo-controlled trials,[18-25] and a meta-analysis [38] that have demonstrated the efficacy of oral desloratadine 5 mg daily in SAR, PAR, IAR, and PER. For example, the percentage of subjects with SAR reporting no or only mild nasal symptoms at the end of a 3-week, open-label study of desloratadine 5 mg/d increased from 42.9% at baseline to 95.2% for sneezing/nasal itching, 40.5 to 94.4% for rhinorrhea, 33.7 to 90.7% for nasal congestion, 70.4 to 97.7% for ocular redness, and 59.0 to 95.9% for burning/itching eyes [26]. In a 4-week randomized clinical trial in patients with PAR, mean changes in total, nasal, and non-nasal symptom scores were significantly (*P *≤ 0.04) greater with desloratadine versus placebo (3.9 vs 3.2, respectively) [22].

A large, multinational program of studies of desloratadine 5 mg/d in IAR and PER found significantly (*P *< 0.05) greater reductions in reflective and instantaneous total and individual symptom scores, including rhinorrhea, nasal congestion, sneezing, and nasal and ocular pruritus [18,19]. Therapeutic response was also significantly (*P *< 0.001) greater with desloratadine than with placebo.

In the current study, nasal airflow significantly increased as measured objectively by PNIF. PNIF provides an inexpensive, uncomplicated, and safe way to assess nasal airflow in patients who have nasal congestion as a result of AR. The improvements observed in this study are consistent with objective measures of nasal airflow recorded in other studies [22,23,39-41]. One multicenter, randomized trial found a statistically significant (*P *= 0.03) increase in morning PNIF with desloratadine 5 mg daily compared with placebo [22]. A second multicenter, randomized trial observed notable, but not statistically significant, improvement in PNIF scores from baseline through day 14 with desloratadine versus placebo [39].

Nasal congestion has been associated with sleep disruption, daytime fatigue, shortness of breath, and headache [13]. Indeed, patients often report that congestion is their most bothersome AR symptom,[9] especially those with persistent or perennial disease,[13] making diagnosis and treatment of paramount importance in improving quality of life. The ARIA guidelines recommend intranasal corticosteroids as first-line treatment for patients with AR when nasal congestion is the predominant symptom [16]. However, a growing and consistent body of evidence from nasal challenge and clinical studies has found that second-generation antihistamines such as desloratadine are also effective in relieving nasal congestion [22,23,25,41-47]. Furthermore, a 2007 meta-analysis that evaluated the efficacy of desloratadine for AR symptoms in 5 allergen-challenge and clinical-setting studies (N = 438) that included nasal congestion measured objectively found significant (*P *= 0.005) improvement in nasal airflow with desloratadine compared with placebo [38].

AR is a worldwide problem with allergenic triggers that differ from region to region. Earlier open-label, practice based clinical trials extended real-world clinical experience with desloratadine for the treatment of AR to such diverse populations as those in Hungary, Israel, Malaysia, the Philippines, Portugal, and Saudi Arabia [29,32,33,35-37]. Subjects with AR treated with desloratadine 5 mg once daily in these trials showed significant improvement in ocular and nasal symptoms, especially congestion, after 2 to 4 weeks of treatment. The present investigation expands that clinical experience to Arab and Asian subjects with SAR or PAR who reside in the Middle East Gulf region.

Desloratadine was safe and well tolerated during the 14-day observation period. No adverse events were reported, and no subjects discontinued treatment because of adverse events.

The observational design of the present study may be considered a limitation. However, this type of study closely approximates the everyday clinical experience of physicians. It is also possible that the improvement in AR symptoms observed over the course of the 14-day study may have abated independent of treatment.

In conclusion, oral desloratadine 5 mg once daily for 14 days relieved nasal and ocular symptoms and improved nasal airflow in a large Arab and Asian population from the Middle East Gulf states of Bahrain, Kuwait, Oman, Qatar, and UAE. These results add to the expanding body of evidence demonstrating that desloratadine is safe and efficacious in diverse populations.

## End Note

Supported by Merck, Sharp & Dohme, Gulf, formerly Schering-Plough A.G., a subsidiary of Merck & Co., Inc., provided financial support to the conduct of the study. Dr Adham is responsible for the work described in this paper. He was involved in the conception and design of the study, the interpretation of data, and revising the manuscript for important intellectual content. He provided final approval of the version to be published.

Merck, Sharp & Dohme, Gulf, formerly Schering-Plough A.G., a subsidiary of Merck & Co., Inc., provided financial support to the conduct of the study. Dr Adham reports no additional conflicts of interest.

The study results included in this article were presented at the XXVII European Academy of Allergy and Clinical Immunology Annual Meeting in Barcelona, Spain, 7-11 June 2008.
